# A Novel Substrate-Based HIV-1 Protease Inhibitor Drug Resistance Mechanism

**DOI:** 10.1371/journal.pmed.0040036

**Published:** 2007-01-16

**Authors:** Monique Nijhuis, Noortje M van Maarseveen, Stephane Lastere, Pauline Schipper, Eoin Coakley, Bärbel Glass, Mirka Rovenska, Dorien de Jong, Colombe Chappey, Irma W Goedegebuure, Gabrielle Heilek-Snyder, Dominic Dulude, Nick Cammack, Lea Brakier-Gingras, Jan Konvalinka, Neil Parkin, Hans-Georg Kräusslich, Francoise Brun-Vezinet, Charles A. B Boucher

**Affiliations:** 1 Eijkman Winkler Center, Department of Medical Microbiology, University Medical Center Utrecht, Utrecht, Netherlands; 2 APHP-Hopital Bichat, Laboratoire Virologie, Paris, France; 3 Monogram Biosciences, San Francisco, California, United States of America; 4 Department of Virology, University of Heidelberg, Heidelberg, Germany; 5 Institute of Organic Chemistry and Biochemistry, Academy of Science of the Czech Republic, Prague, Czech Republic; 6 Roche Bioscience, Palo Alto, California, United States of America; 7 Department of Biochemistry, Université de Montreal, Montreal, Canada; McGill University AIDS Centre, Canada

## Abstract

**Background:**

HIV protease inhibitor (PI) therapy results in the rapid selection of drug resistant viral variants harbouring one or two substitutions in the viral protease. To combat PI resistance development, two approaches have been developed. The first is to increase the level of PI in the plasma of the patient, and the second is to develop novel PI with high potency against the known PI-resistant HIV protease variants. Both approaches share the requirement for a considerable increase in the number of protease mutations to lead to clinical resistance, thereby increasing the genetic barrier. We investigated whether HIV could yet again find a way to become less susceptible to these novel inhibitors.

**Methods and Findings:**

We have performed in vitro selection experiments using a novel PI with an increased genetic barrier (RO033-4649) and demonstrated selection of three viruses 4- to 8-fold resistant to all PI compared to wild type. These PI-resistant viruses did not have a single substitution in the viral protease. Full genomic sequencing revealed the presence of NC/p1 cleavage site substitutions in the viral Gag polyprotein (K436E and/or I437T/V) in all three resistant viruses. These changes, when introduced in a reference strain, conferred PI resistance. The mechanism leading to PI resistance is enhancement of the processing efficiency of the altered substrate by wild-type protease. Analysis of genotypic and phenotypic resistance profiles of 28,000 clinical isolates demonstrated the presence of these NC/p1 cleavage site mutations in some clinical samples (codon 431 substitutions in 13%, codon 436 substitutions in 8%, and codon 437 substitutions in 10%). Moreover, these cleavage site substitutions were highly significantly associated with reduced susceptibility to PI in clinical isolates lacking primary protease mutations. Furthermore, we used data from a clinical trial (NARVAL, ANRS 088) to demonstrate that these NC/p1 cleavage site changes are associated with virological failure during PI therapy.

**Conclusions:**

HIV can use an alternative mechanism to become resistant to PI by changing the substrate instead of the protease. Further studies are required to determine to what extent cleavage site mutations may explain virological failure during PI therapy.

## Abstract 2

Changes in the cleavage site of the Gag substrate for the HIV protease can convey resistance to protease inhibitors and might contribute to virologic failure during therapy that includes these drugs.

## Editors' Summary

**Background.:**

Twenty-five years ago, infection with the human immunodeficiency virus (HIV)—the causative agent of AIDS—was a death sentence. However, drugs that attack various stages of the HIV life cycle were soon developed that, although not curing the infection, kept it in check when used in combination and greatly increased the life expectancy of people infected with HIV. Unfortunately, viruses resistant to these drugs have rapidly emerged and antiviral therapy now fails in many patients. The use of HIV protease inhibitors (PIs) in combination therapies, for example, has led to the stepwise selection of viral variants resistant to these drugs. Resistance is first acquired when the viral protease changes so that PIs no longer bind to it and inhibit it efficiently. These changes often reduce the efficiency with which the protease binds its substrates—polyproteins called Gag and GagPol that it chops up into smaller proteins to make new viral particles. So the next step is the accumulation of changes elsewhere in the protease that make it work better, and sometimes changes in its substrate that make it easier to cut; these compensatory changes do not directly affect viral resistance to PIs.

**Why Was This Study Done?:**

To prevent viruses with resistance to PIs emerging, drug doses are kept high in patients and new PIs are being developed with high potency against known PI-resistant HIV variants. Both approaches set a “high genetic barrier” to the development of PI resistance by ensuring that HIV has to incorporate many changes in its protease to become resistant. But, the HIV genome naturally changes—mutates—very rapidly, so novel HIV variants could emerge that are less susceptible to the new potent PIs without the virus having to leap this high genetic barrier. In this study, the researchers have investigated whether HIV can find an alternative route to PI resistance that does not involve the introduction of multiple changes into its protease.

**What Did the Researchers Do and Find?:**

The researchers took wild-type HIV and treated it in the laboratory with a new PI regimen that has a high genetic barrier. By gradually increasing its concentration, the researchers selected three viral populations that were able to grow in 4- to 8-fold higher concentrations of the PI than wild-type virus. None of these populations had mutations in the viral protease. Instead, they all had mutations near one of the sites—the NC/p1 site—where the protease normally cuts the Gag polyprotein. These mutations, the researchers report, enhanced the overall efficiency with which the wild-type protease cleaved the polyprotein, and a selection experiment with another PI showed that the development of PI resistance through alterations near the NC/p1 cleavage site was not unique to one PI. The researchers also investigated the potential clinical significance of this new drug resistance mechanism by looking for the same mutations in nearly 30,000 patient samples. Many of the samples did indeed have these mutations. Finally, they showed that mutations at the NC/p1 cleavage site were associated with virological failure (increased viral replication) during PI therapy in an ongoing clinical trial.

**What Do These Findings Mean?:**

These results suggest that increased polyprotein processing because of mutations in the natural substrate of the HIV protease might be a new mechanism by which HIV can become resistant to PIs. This strategy, which occurs in the laboratory and in patients, allows HIV to develop PI resistance without the need for multiple changes in its protease and so avoids the high genetic barrier to resistance that new PIs provide. Clinical studies are now needed to test which of the mutations seen in this study contribute to virological failure, whether the degree of this failure is clinically relevant, and whether these substrate mutations enhance the effect of protease mutations. If the clinical importance of the new mechanism is confirmed, genetic examination of both the polyprotein and the protease will be needed when trying to figure out why a PI-containing therapy is failing in individual patients. Furthermore, it will be necessary to test whether this mechanism can contribute to the development of resistance when evaluating new drugs.

**Additional Information.:**

Please access these Web sites via the online version of this summary at http://dx.doi.org/10.1371/journal.pmed.0040036.

- US National Institute of Allergy and Infectious Diseases factsheet on HIV infection and AIDS
- US Department of Health and Human Services information on AIDS
- US Centers for Disease Control and Prevention information on HIV/AIDS
- Aidsmap information on HIV and AIDS provided by the charity NAM
- BioAfrica, Bioinformatics for HIV Research, information on HIV-1 protease cleavage sites

## Introduction

Selection of drug-resistant viruses is a major problem causing therapy failure in a substantial proportion of patients infected with HIV-1. Increasingly, these drug-resistant viruses are transmitted and reported to persist in the absence of therapy [1–4]. Moreover, the use of sequential monotherapy in developed countries has led to the emergence of HIV variants that are resistant to one or more classes of antivirals.

The introduction of protease inhibitors (PIs) has been one of the key components in the success of combination therapy. The viral protease is required for the cleavage of the precursor Gag and GagPol polyproteins, resulting in mature infectious virus particles. The genetic basis of resistance to PIs has been well documented in the last decade. The development of PI resistance is usually a stepwise process [5–7]. A change in the substrate-binding pocket of protease is thought to be the first step, leading to decreased binding of the inhibitor (and hence to resistance), as well as decreased binding of the natural substrate and thus affecting virus replication [8–11]. To compensate for this, additional mutations accumulate within the protease sequence that have minor effects on resistance, but improve the processing efficiency of the resistant enzyme, thus restoring (at least partially) viral replication [11–14]. Besides the genetic alterations in the protease sequence, compensatory mutations have been described in the viral Gag polyprotein, most commonly found in the vicinity of the actual cleavage sites. The mutations are thought to adapt the virus to the altered substrate-binding cleft of the mutant enzyme [14–18]. Therefore, substrate alterations are generally believed to be compensatory changes that do not directly affect protease resistance.

Two approaches have been developed to prevent PI resistance. The first approach is to increase the level of PI in the plasma of the patient by combining them with low-dose ritonavir, a cytochrome P450 3A4 enzyme inhibitor [19]. As a consequence of this, drug levels are higher and the virus needs to acquire more mutations in the protease sequence to achieve clinical resistance. The second approach is to develop novel HIV PIs with high potency against known PI-resistant HIV variants [20–22]. Both approaches require a considerable increase in the number of protease mutations necessary to achieve clinical resistance, thereby increasing the genetic barrier.

It should not come as a surprise that HIV would use alternative resistance mechanisms that could defeat the strategy of imposing a high genetic barrier. Indeed, a clinical trial using the potent PI lopinavir boosted with ritonavir in therapy-naive patients, and thus exploiting a high genetic barrier approach, revealed virological failure in 20% of patients [23]. In this study, failure could not be attributed to the development of traditional protease resistance mutations, as none of the viruses obtained from patients failing treatment displayed changes in the viral protease sequence, thus suggesting a novel resistance mechanism. We have performed clinical and laboratory studies to test whether HIV can indeed become resistant to PI without changing the viral protease.

## Methods

### Viral Culture

#### Cells.

SupT1 and MT-2 cells were maintained in RPMI 1640 medium with L-glutamine (BioWhittaker, http://www.cambrex.com) supplemented with 10% fetal bovine serum (FBS; Gibco, http://www.invitrogen.com) and 10 μg/ml gentamicine (Gibco). 293T cells were maintained in Dulbecco modified Eagle medium (BioWhittaker) supplemented with 10% FBS and 10 μg/ml gentamicin. All cells were passaged twice weekly.

#### In vitro selection experiments.

Multiple in vitro selection experiments were performed using the HIV-1 reference strain HXB2 in the presence of increasing concentrations of RO033-4649 or ritonavir. The selection was initiated by infection of SupT1 cells (MOI 0.01) in the absence of drug. After a 1-h incubation at 37 °C, culture medium supplemented with the drug was added to a final volume of 10 ml. During the experiment the culture was monitored daily for the appearance of syncytia. When full-blown syncytia were observed, cell-free virus was used to initiate the next cell culture passage. The RO033-4649 concentration was raised during the subsequent passages from 20 nM (IC_50_ of HXB2) to 100 nM (IVS-32) and 120 nM (IVS-1 and IVS34; passage 1: 20 nM; passage 2: 40 nM; passage 3: 60 nM; passages 4 and 5: 70 nM; passages 6 and 7: 80 nM; passages 8 and 9: 100 nM; and passage 10: 120 nM). The ritonavir concentration was raised from 60 nM (IC_50_ of HXB2) to finally 960 nM (passage 1: 60 nM; passage 2: 120 nM; passage 3: 240 nM; passages 4 and 5: 480 nM; and passage 6: 960 nm). HIV-1 RNA from the virus supernatant of the passage with the highest drug concentration was used for genotypic analysis of the complete viral genome.

#### Drug susceptibility analysis.

The infectious virus titre (TCID_50_) was determined using endpoint dilutions in MT2 cells. Drug susceptibility of the viruses was determined in duplicate using the multiple-cycle MTT assay [24]. In addition, drug susceptibility was determined using a single-cycle assay by Monogram Biosciences (http://www.monogrambio.com) using the PhenoSense assay [25].

### Viral RNA Analysis

#### Amplication and sequencing of the viral genome.

Viral RNA was isolated using the Nuclisens Isolation Kit (Organon Teknika, http://www.biomerieux-usa.com). Briefly, a 100 μl sample was mixed with 900 μl lysis buffer and 50 μl silica and incubated for 10 min at room temperature to allow binding of the nucleic acid to the silica particles. Unbound material was removed by several washing steps, after which the RNA was eluted in 100 μl of 40 ng/μl polyA RNA.

The extracted HIV-1 RNA was then used to reverse transcribe and amplify the viral genome, essentially as described before [13] (Table S1). All PCR-amplified products were purified using the QIAquick PCR purification kit (Qiagen, http://www.qiagen.com).

Subsequently, population sequencing was performed using the BigDye Terminator v1.1 Cycle Sequencing Kit (Applied Biosystems, http://www.appliedbiosystems.com) (Table S1).

### Generation of Recombinant Virus

The viral DNA fragment including the C-terminal portion of Gag (p2-NC-p1-p6) and protease was cloned into a HXB2 reference strain as previously described [26]. As a consequence, the recombinant virus also acquired the wild-type protease sequence from the in vitro selection experiment. Therefore, the PCR product encompassing the C-terminal portion of Gag and protease and the vector (pHXB2ΔCSPR) were digested with BstEII and MluNI. PCR product and vector (pHXB2ΔCSPR) were ligated using the Rapid DNA Ligation Kit (Roche Diagnostics http://www.roche-diagnostics.com).

Subsequently, the isolated plasmid was used to transfect 293T cells. Therefore, 5–6 × 10^6^ 293T cells were seeded the day prior to transfection to achieve 90%–95% confluence on the day of transfection. For transfection of 10 μg of plasmid DNA, Lipofectamine 2000 (Invitrogen, http://www.invitrogen.com) was used according to the manufacturer's protocol. The transfection recombinant virus was harvested 2 d later.

Sequence analysis confirmed the generation of a recombinant HIV Gag clone of IVS-1 containing the K436E+I437T amino acid substitution. In the original virus population of IVS-1, a mixture at amino acid R464R/K in p6^gag^ was present, and a virus clone containing the wild-type codon at position 464 was picked. The recombinant HIV Gag clone of IVS-32 contained the I437V amino acid change. The recombinant HIV Gag clone of IVS-34 contained the I437T and an A15T change in p6^pol^. Since the IVS-34 virus stock contained the K43T amino acid change in the viral protease, a virus stock from an earlier passage was used for the generation of this particular clone.

### Viral Frameshift Analysis

#### Dual-luciferase constructs.

A dual-luciferase reporter plasmid was used as described in Dulude et al. [27], based on previously published systems [28,29]. In short, the HIV-1 frameshift region (nucleotides 2062 to 2139 of the *HXB2* genome) was inserted between the coding sequence of the *Renilla* luciferase and the firefly luciferase gene in an appropriate vector derived from pcDNA3.1. In this pDual-HIV(−1) construct, only the ribosomes that initiate translation at the initiator codon for the *Renilla* luciferase but change the reading frame by one base in the 5′ direction in the frameshift region synthesize the firefly luciferase, which is thus fused to *Renilla* luciferase. A control plasmid, pDual-HIV(0), was used to measure the translation level of an in-frame firefly luciferase coding sequence relative to that of the *Renilla* luciferase. Derivatives mutated in the frameshift region were generated by PCR amplification of the parental pDual-HIV(−1) construct with the following forward and reverse mutagenic primers: 5′-CAGGGGGTACCGGATTGTACTGAGAGACAGGCTAATTTTTTAGGGN_1_AGN_2_N_3_CTGGCC-3′ and 5′-CCACTTGTAGAGCATGCGCCTTA-3′, which contains a KpnI and a Pfl23II restriction site (underlined), respectively. Nucleotides at positions N_1_ = A, N_2_ = A, and N_3_ = T in the wild-type HXB2 sequence were changed to N_1_ = G and N_3_ = C in mutant IVS-1, N_2_ = G in mutant IVS-32, and N_3_ = C in mutant IVS-34. The digested PCR products were then inserted between the KpnI and Pfl23II restriction sites of the wild-type pDual-HIV(−1) digested with the same enzymes.

#### Frameshift assays.

The effect of the mutations in the HIV-1 frameshift region on the −1 frameshift efficiency was measured using the dual-luciferase reporter plasmids described above. Frameshifting was monitored by transient transfection into 293FT cells of the dual-luciferase vectors (Invitrogen). The day before transfection, 2 × 10^4^ cells/well were seeded in 24-well plates and maintained in Dulbecco's modified Eagle medium (DMEM, Gibco) supplemented with 10% fetal bovine serum (FBS, Wisent). Briefly, 0.5 μg of each dual-luciferase reporter construct was transfected into cells using a standard calcium-phosphate precipitation method [30], and cells were grown for 48 h before being harvested. Cells were washed twice with PBS and lysed with 100 μl of the Cell Passive Lysis Buffer (Promega, http://www.promega.com). The firefly versus the *Renilla* luciferase activities of each construct were measured as relative light units with a Berthold Lumat LB 9507 luminometer (http://www.bertholdtech.com), using a Dual-Luciferase Reporter Assay System kit from Promega. Frameshift efficiencies were determined by dividing the ratio of the firefly to *Renilla* luciferase activities from the wild-type or mutant constructs to the firefly to *Renilla* luciferase ratio from the in-frame pDual-HIV(0) control construct.

### Western Blot Analysis

#### Transfection.

293T cells were transfected with recombinant proviral plasmids by the calcium-phosphate method, and PI was added at the time of transfection where indicated. Culture medium and cells were harvested after 48 h, and virus particles were collected from cleared media by centrifugation at 44,000 rpm in a TLA 45 Rotor (Beckman Coulter, http://www.beckmancoulter.com) for 1 h at 4 °C.

#### Western blotting.

For Western blot analysis, samples were boiled in SDS sample buffer, separated by 12.5% or 15% SDS-PAGE, and transferred to a nitrocellulose membrane. Protein detection was performed following incubation with antibodies against reverse transcriptase (RT), capsid (CA), or nucleocapsid (NC) and appropriate secondary antibodies using the Super Signal West detection kit (Pierce, http://www.piercenet.com) according to the manufacturer's protocol. Quantitative Western blots were performed using the Odyssey Infrared Imaging System as recommended by the manufacturer (LI-COR, http://www.licor.com).

### Analysis of RT Activity

Virus supernatant from 293T cells transfected with wild-type HXB2 or 436E+437T constructs was used for the detection of RT activity using the colorimetric Reverse Transcriptase Assay (Roche Diagnostics) according to the manufacturer's protocol.

### Peptide Cleavage Analysis

Peptide cleavage experiments were performed in triplicate at pH 5.5, 37 °C, using 750 nM of wild-type protease and 200 μM substrate, and were analyzed on an HPLC Symmetry C18 column using a gradient of methanol in water. The reactions were stopped after 1 h by adding trifluoroacetic acid (TFA) to a final concentration of 2%.

### Database Analysis

The prevalence and drug susceptibility of the 431, 436, and 437 mutations in clinical samples was determined among clinical samples submitted to Monogram Biosciences (formerly ViroLogic, Inc.) for routine combination phenotype/genotype testing using PhenoSense HIV and GeneSeq HIV [31]. These assays include amplification by RT-PCR of the C-terminal 82 amino acids of Gag as well as protease and RT from the patient viruses. Phenotypic drug susceptibility (fold change in IC_50_) of these recombinant viruses for the PIs amprenavir, indinavir, nelfinavir, ritonavir, saquinavir, lopinavir, and atazanavir was determined. Nucleotide sequences were determined by di-deoxy chain terminator sequencing. All samples for which unambiguous Gag sequence could be determined were included in the analysis. Mixtures containing wild-type and mutant variants were scored as mutant. Primary protease resistance-associated mutations were defined as any change versus wild-type at positions 23, 24, 30, 32, 46, 47, 48, 50, 54, 82, 84, 88, and 90 in the viral protease, with the following exceptions: I54V and N88D (not reported to occur without other primary mutations) and V82I (known polymorphism in PI-naive patients).

### Investigation of the Association between Gag Substitutions and Virological Response in the NARVAL Trial

Baseline specimens from 203 highly active antiretroviral therapy–experienced participants in the NARVAL trial (ANRS 088) were sequenced with regard to the HIV protease and C-terminal part of Gag [32]. Virological response (defined as mean change in viral load between entry and week 12 of the treatment) were compared according to the presence or absence of mutations at codons 431, 436, and 437 of HIV Gag by using the nonparametric Mann-Whitney or Kruskall-Wallis test (*p* < 0.05 was considered to indicate statistical significance) in univariate analyses. Mutations for which the *p* value was lower than 0.05 were retained and then analyzed in a linear multivariate regression in order to assess whether or not it was an independent predictor of the virological response (mean change from baseline in viral load). The independent variables used in the model were the variables predictive of response in the overall multivariate analysis of the NARVAL trial [33], that is to say prescription of efavirenz in non-nucleoside reverse transcriptase inhibitor–naive patients, baseline prescription of lamivudine (3TC), baseline prescription of abacavir (ABC) in ABC-naive patients, baseline viral load, previous PI exposure for more than 30 mo, number of nucleoside-associated mutations ≥3, and randomization arm (SOC versus P). In addition, the model was also adjusted on use of nelfinavir at baseline and on the number of PI mutations (L10IRVF, K20RM, M36I, A71VT, G73SA, V82AFTS, L90M) found to be associated with the virological response in univariate analyses in this subset of patients. Calculations were performed with the SPSS software package (SPSS, http://www.spss.com).

## Results

### In Vitro Selection of HIV Variants Resistant to the PI RO033-4649

**Figure 1:**
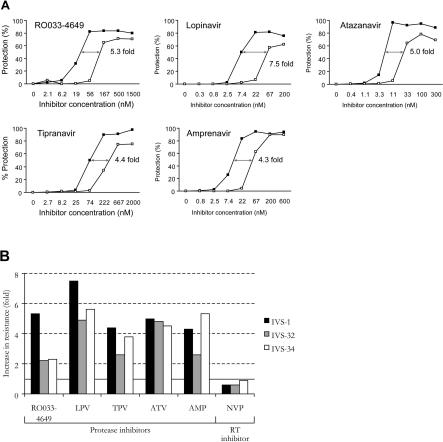
Determination of Phenotypic Drug Susceptibility of the In Vitro-Selected Virus Populations (A) Investigation of phenotypic drug susceptibility of IVS-1 (open squares) as compared to wild-type HIV (HXB2; filled squares) to the protease inhibitors RO033-4649, lopinavir, tipranavir, atazanavir, and amprenavir. Fold increases in IC_50_ are indicated. (B) Representation of the fold increases in phenotypic drug resistance of IVS-1, IVS-32, and IVS-34 as compared to wild-type HIV (HXB2) to the PI R0033–4649, lopinavir (LPV), tipranavir (TPV), atazanavir (ATV), and amprenavir (AMP), and as a control to the RT inhibitor nevirapine (NVP).

The novel PI RO033-4649 with a high genetic barrier was used to investigate the potential of alternative drug resistance pathways in vitro [20]. This substrate-based inhibitor has an IC_50_ value of about 20 nM for wild-type virus. Multiple selection experiments (*n* = 36) using an HIV-1 reference strain (HXB2) were performed in the presence of very slowly increasing concentrations of the PI. Only three individual selection experiments out of 36 (IVS-1, IVS-32, and IVS-34) resulted in viral populations that were able to replicate in the presence of high concentrations of RO033-4649 (≥100 nM), indicating that the novel PI exerted a strong selective pressure. Phenotypic tests were performed to investigate the resistance profiles of these viruses. Virus IVS-1 demonstrated a 5.3-fold increase in IC_50_ for RO033-4649 (Figure 1A and 1B), whereas the two other viruses demonstrated a 2- to 3-fold increase in IC_50_ (Figure 1B). Moreover, comparable and even higher levels of resistance (up to 8-fold) were observed for four clinically used PIs, lopinavir, tipranavir, atazanavir, and amprenavir (Figure 1A and 1B). In a control assay no change in susceptibility to a non-nucleoside RT inhibitor (nevirapine) was observed (Figure 1B).

### Determination of Genetic Correlates of Protease Resistance

To identify the genotypic changes associated with PI resistance, we sequenced the protease-coding region of all three viruses. No amino acid changes in the viral protease sequence were observed, with the exception of a single K43T change in IVS-34. This substitution, when introduced into a wild-type reference strain, did not cause an increase in resistance to RO033-4649 (IC_50_ of 27 nM for both wild-type and the K43T mutant).

**Figure 2:**
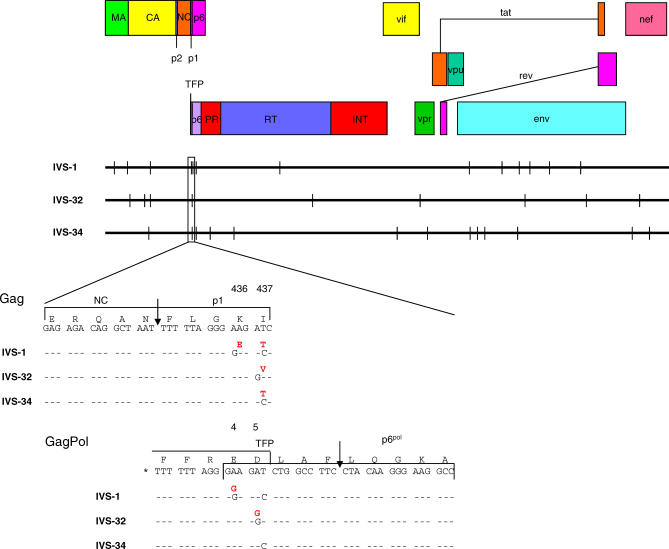
Schematic Representation of the Distribution of all Amino Acid Changes Appearing during in vitro Selection Experiments Using RO033-4649 Horizontal bars below the gene organization scheme represent the genomes of viruses from the three in vitro selection experiments: IVS-1, IVS-32, and IVS-34. Vertical lines illustrate the observed amino acid changes. The C-terminal portion of Gag was expanded to precisely map the nucleotide changes leading to amino acid changes (bold) in both translational reading frames (Gag and GagPol). In addition, the three different cleavage sites (NC/p1, NC/TFP, and TFP/p6^pol^) are indicated. The control experiments, in which no drug was added during the in vitro selections, demonstrated no amino acid changes in the viral Gag and protease region.

The observation that PI resistance did not map to the protease-coding region suggested that mutations in alternative regions were responsible for the observed phenotype. To identify these mutations, the full-length genome of all three viruses was sequenced (Figure 2). Mutations unique to each viral population were scattered throughout the three genomes. Interestingly, all three viruses contained mutations in the C-terminal region of the viral *gag* gene at amino acid position 437 in the p1-coding region of *gag* (I437T/V). In IVS-1 there was an adjacent K436E amino acid change. Two of the viruses demonstrated selection of amino acid changes in the overlapping reading frame (p6^pol^) as well.

**Figure 3:**
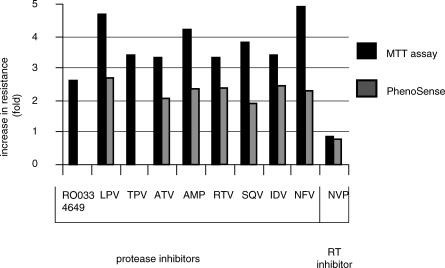
Determination of Phenotypic Drug Susceptibility of C-Terminal Gag Clones Representation of the fold increases in phenotypic drug susceptibility of the C-terminal Gag clone derived from IVS-1, containing the 436E+437T amino acid change as compared to wild-type HIV (HXB2). The black bars indicate the fold increase as determined in the multiple-cycle MTT drug susceptibility assay, and the gray bars indicate the fold increase as determined in the single-cycle PhenoSense drug susceptibility assay. Drug susceptibility to the PI RO033-4649, LPV, TPV, ATV, AMP, ritonavir (RTV), saquinavir (SQV), indinavir (IDV), and nelfinavir (NFV), and as a control to the RT inhibitor NVP was determined. RO033-4649 and TPV were not available at the time of PhenoSense assay testing.

To determine whether the changes in *gag* alone could cause the reduced susceptibility to PI, we cloned the C-terminal portion of *gag* (p2-NC-p1-p6) and protease into the reference strain HXB2. This resulted in the generation of three clones containing the following changes compared to wild-type HXB2: IVS-1 (436E+437T), IVS-32 (437V), and IVS-34 (437T and A15T in p6^pol^). Resistance profiles for these recombinant viruses confirmed that the genotypic changes observed in Gag caused part of the initially observed reduced PI susceptibility. This was most obvious for the combination of 436E+437T (IVS-1), which displayed a 2- to 5-fold increase in IC_50_ to all PIs in a multiple-cycle drug resistance analysis and a 2- to 3-fold increase in a single-cycle assay (Figure 3). The single substitutions caused an up to 2-fold increase in IC_50_ in the single-cycle assay.

### Identification of the Underlying Resistance Mechanism

The observed resistance to various PIs could be caused by two different mechanisms. First, since the mutations are located in the region that regulates Gag–GagPol ribosomal frameshifting, the ratio of Gag to GagPol proteins in infected cells could be altered in favor to increased levels of protease. Second, since the amino acid changes are located near three cleavage sites (NC/p1, NC/TFP, and TFP/p6pol), the polyprotein processing rates could be altered.

**Figure 4:**
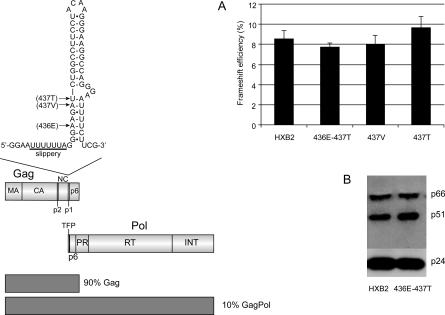
Schematic Representation of the Viral Frameshift Region and Investigation of Frameshift Efficiency The RNA structure of the frameshift stimulatory signal is represented here [30,51]. The nucleotide and amino acid changes (between brackets) as observed in our in vitro selection experiments are indicated. (A) The relative frameshift efficiency of wild-type HIV (HXB2) and the p1 constructs of IVS-1 (436E+437T), IVS-32 (437V), and IVS-34 (437T). The values of the frameshift efficiency are the means of four independent experiments, with bars representing the standard errors. (B) Analysis of differences in levels of HIV GagPol products (RT, p66, and p51) as compared to HIV Gag products (CA, p24) by Western blot analysis.

Several approaches were used to investigate whether an increase in the efficiency of frameshifting, resulting in increased levels of HIV protease, could explain the resistant phenotype (Figure 4). Inspection of the positions of the mutations with respect to the viral frameshifting signal suggested that such a mechanism would be unlikely (Figure 4). Experimentally, the effect of the observed mutations was evaluated in a frameshift efficacy assay using two reporter genes, such that expression of the second reporter depends on ribosomal frameshifting. The measured frameshift efficiencies for the p1 mutant constructs were not significantly different from those observed for the wild-type construct (HXB2, 8.55% ± 0.84%; 436E+437T, 7.7% ± 0.42%; 437V, 7.94% ± 0.96%; and 437T, 9.68% ± 1.11%; Figure 4A). An increase in frameshifting would result in increased levels of all Pol proteins, including RT. We therefore investigated the amount of viral RT in HXB2 as compared to the p1 mutant 436E+437T by Western blot analysis (Figure 4B). In line with the frameshift assay results, no sign of increased RT levels was observed; this was confirmed by analysis the levels of RT activity (normalized based upon levels of p24 [436E+437T, 104% RT activity relative to HXB2]). In conclusion, all data showed that the PI resistance phenotype was not caused by increased frameshift efficacy.

**Figure 5:**
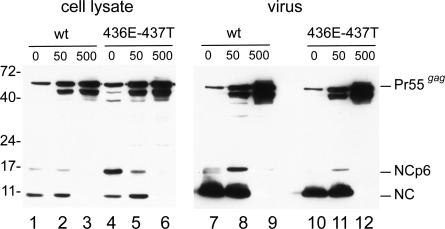
Analysis of Gag Polyprotein Processing Western blot analysis of cell lysates (left panel) or virus particles (right panel) obtained by ultracentrifugation of cell culture supernatants from 293T cells transfected with the respective recombinant virus plasmids in the absence of inhibitor or in the presence of 50 or 500 nM of RO033-4649. Molecular mass standards are depicted on the left, Gag-derived proteins are identified on the right. Protein detection was performed following incubation with a specific antibody against NC.

The C-terminal Gag region contains three protease cleavage sites (NC/p1, NC/TFP, and TFP/p6pol). To determine whether altered cleavage could explain the observed phenotype, we investigated the overall proteolytic processing and specifically the processing of the C-terminal part of Gag. Since the 436 and 437 mutations are located distantly from the actual cleavage site we decided to concentrate on cleavage of the complete Gag polyprotein rather than analysis of mutant decapeptides. To this end, 293T cells were transfected with either a wild-type HXB2 proviral clone or the 436E+437T mutant construct in the presence or absence of RO033-4649. Cell and particle lysates were analyzed by Western blotting using antisera against CA or NC (Figure 5, Table 1, and Figure S1). No major difference in overall processing was observed. Specific antiserum against NC showed that the fully cleaved NC was detected as the main product for wild-type and mutant virus–infected cells and virion preparations with a shift to the uncleaved precursor at increasing PI concentrations (Figure 5). A notable difference was observed, however, for the intermediate-processing product NCp6 (containing the NC, p1, and p6 regions). In mutant transfected cells, this product was more prominent than in wild-type transfected cells, while the opposite was observed for virion preparations (Figure 5). NCp6 was almost undetectable in virus preparations from mutant 436E+437T–infected cells, while it was clearly detectable in wild-type virus (Figure 5; compare lanes 7 and 10). This difference became much more obvious at a concentration of 50 nM RO033-4649 (Figure 5; compare lanes 8 and 11), where a difference in PI sensitivity is actually observed (IC_50_ for wild type, 22 nM; for IVS-1, 53 nM). This effect was observed for virus from four independent transfections and was not due to loading differences since the amounts loaded were normalized by ELISA and quantitative immunoblot. Thus, the NCp6 intermediate was processed more efficiently in maturing viruses of the resistant variant with mutations close to the NC/p1 cleavage site.

**Table 1:**
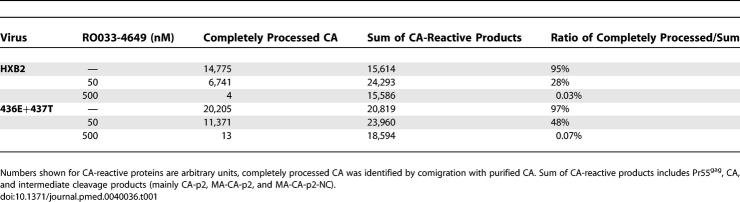
Polyprotein Processing of HIV Wild-Type and Mutant Virus Using a Quantitative Immunoblot Analysis

Besides this effect on cleavage at the site close to the actual mutation, we also observed an effect of the 436E+437T mutation on polyprotein processing at a distant site. Immunoblot analysis of wild-type and mutant transfected cells and virus preparations using anti-CA antiserum again revealed no obvious difference in processing patterns (Figure S1). A clear difference was observed, however, when the relative amount of fully processed CA compared to all CA-reactive products in virion preparations at suboptimal inhibitor concentration was compared by quantitative Western blot analysis (Table 1). Complete processing of CA, including late cleavage of the C-terminally adjacent p2 peptide is essential for CA condensation and viral infectivity [34]. Without an inhibitor, >95% of all CA-reactive antigen was completely processed, both in the case of wild-type and resistance variant, and this ratio changed to <0.1% at the fully inhibitory concentration of 500 nM RO033-4649. At a concentration of 50 nM, on the other hand, 20%–30% of CA was completely processed in the case of the wild-type, while 35%–50% was completely processed in the case of the resistance variant; this difference was again reproducible in several independent experiments. The observed effect was due mostly to a shift from fully processed CA to CA-p2 in the case of wild-type virus (unpublished data). These results indicated that amino acid changes near the NC/p1 cleavage site enhanced not only the processing of the NC-p6 intermediate product but also overall polyprotein processing and thus virus maturation. This effect was most pronounced at a discriminating PI concentration.

### In Vitro Selection Using Ritonavir

To investigate whether selection of genetic alterations close to the protease cleavage sites is a general strategy used by the virus to acquire PI resistance, we performed in vitro selection experiments using the approved PI ritonavir. Multiple selections (*n* = 7) using an HIV-1 reference strain (HXB2) were performed in the presence of increasing concentrations of ritonavir. In all experiments we readily obtained viruses containing known PI resistance–associated mutations in the viral protease. In three of the experiments we observed an A431V amino acid substitution in the NC/p1 cleavage site, combined with known resistance substitutions in protease (V32I, M46I/L, I54V, V82A/F, and I84V). When we analyzed clonal isolates it was found that this cleavage site change occurred without changes in the protease itself in one experiment. This A431V change, when introduced into a wild-type reference strain, conferred 3.8-fold reduced susceptibility to ritonavir (IC_50_ of 7.3 nM [wild-type] as compared to 28 nM [A431V mutant]). This observation suggested that changes at other positions in the NC/p1 cleavage site could also directly cause PI resistance. To test whether the PI resistance conferred by A431V was associated with a change in processing efficiency at this site, we investigated the relative turnover by wild-type protease of a decapeptide corresponding to either the wild-type HXB2 NC/p1 (ERQAN/FLGKI) or the mutant NC/p1 (ERQ**V**N/FLGKI) cleavage site sequence. We observed an increased rate of peptide cleavage for the NC/p1 peptide containing the ritonavir resistance–associated A431V substitution (relative turnover of 0.18 [wild-type] as compared to 0.35 [for the mutant]; Table 2).

**Table 2:**
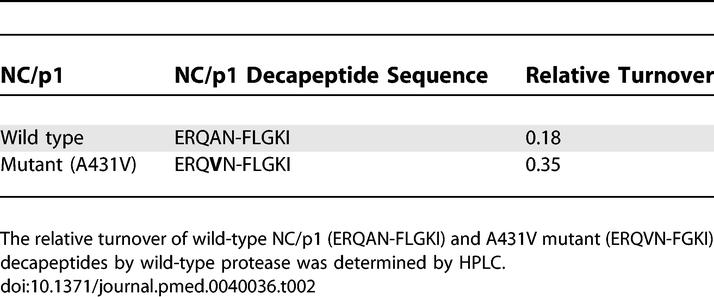
Relative Turnover of Wild-Type and Mutant NC/p1 Decapeptides by Wild-Type Protease

### Potential Clinical Significance of the Novel Drug Resistance Mechanism

To investigate the potential clinical significance of improved cleavage associated with mutations at positions 431, 436, or 437 in HIV Gag, we performed two studies. First, we determined the frequency at which these mutations were found in clinical samples in a database of 28,241 patient samples for which the genotype encompassing the protease, the RT, and the C-terminal 82 amino acids of Gag and the phenotypic measure of drug susceptibility were available. At positions 431, 436, or 437, 13%, 8%, and 10% of the samples had evidence of a mutation, respectively. In samples with no primary PI mutations versus those with one or more such mutations, the percentage of samples with any mutation at positions 431, 436, and 437 was 1% versus 34%, 6% versus 12%, and 6% versus 16%, respectively. Multiple amino acid substitutions were observed at 431 (mostly V and I), 436 (mostly R and G), and 437 (mostly V or L). These changes were observed in viruses from infected patients and are associated with previously “unexplained” PI resistance [31].

Second, we analyzed whether mutations at positions 431, 436, or 437 are associated with virological failure after PI therapy. This was investigated for the NARVAL trial (ANRS 088) [35], a randomized study designed to assess the clinical utility of drug-resistance testing as the basis for choosing a new antiretroviral regimen following highly active antiretroviral therapy failure. We examined the prevalence of substitutions at codons 431, 436, or 437 at baseline (after PI therapy failure) and their association with week-12 virologic response (defined as the mean change in viral load) during subsequent PI containing therapy (Table 3).

**Table 3:**
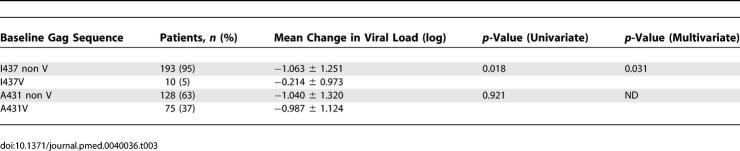
Association between Substitutions at Codons 437 and 431 and Virological Response

This analysis demonstrated a 37% prevalence of the A431V substitution at baseline, which was not associated with lack of virological response during subsequent PI therapy. The I437V substitution, in contrast, which was prevalent in 5% of all baseline isolates, was significantly associated with lack of virological response (*p* = 0.018 in univariate analysis; *p* = 0.031 in multivariate analysis). Changes at codon 436 (K436R/N) were present in only a few patient isolates and could not be evaluated. Of the clinical samples demonstrating a I437V substitution, 60% also presented a primary protease mutation at position 82 (*p* = 0.020), which indicates that this particular Gag mutation is also associated with a reduced virological response when present in combination with mutations in protease itself.

## Discussion

In this study, we show that increased polyprotein processing due to mutations in the natural substrate rather than the enzyme itself represents a novel mechanism by which HIV-1 develops resistance to PIs.

To prevent the widespread development of HIV PI resistance, novel approaches have been developed. These approaches have in common that multiple amino acid changes in the viral protease sequence are required to give clinical resistance. We demonstrate that HIV can escape this strategy by selection of amino acid changes near the NC/p1 cleavage site, resulting in increased cleavage and PI resistance in the absence of any protease mutation. Moreover, selection experiments using an approved PI demonstrated that enhanced processing at this site is a common principle in resistance to PI. Furthermore, we demonstrate that this novel mechanism is not only an in vitro observation, but may have clinical relevance. First, the same mutations that confer in vitro increased NC/p1 cleavage were also observed in vivo. Moreover, in clinical samples lacking primary protease mutations, both the 431V and the 437V mutation were significantly associated with a more than 5-fold increase in resistance to at least one clinically used PI [31]. This suggests that these changes at the NC/p1 cleavage site can affect susceptibility to PIs independently from recognized mutations in PR. Second, we show that enhanced processing of the NC/p1 cleavage site is associated with virological failure during PI therapy.

Development of PI resistance is believed to be due to a stepwise accumulation of amino acid changes in the target protein of the drug, the viral protease [5–7]. These mutations are located in the substrate-binding pocket or at more distant sites in the enzyme and directly or indirectly reduce the affinity of protease for the inhibitor, but also affect binding of its natural substrate. Mutations close to the cleavage sites in the viral Gag and GagPol proteins were considered to be compensatory changes that restore binding of the mutant enzyme and thus viral fitness [14–17].

Previous studies have identified an association between the selection of protease mutations and the appearance of mutations in cleavage sites [36–38]. Changes at the p1/p6 cleavage site (L449F or P453L), which on their own do not confer resistance, were associated with reduced sensitivity in the background of the I50V in protease [18,39]. In this study, we demonstrate the selection of substitutions in the Gag substrate without any preceding alterations in the viral PR sequence itself. These cleavage site changes on their own contribute directly to PI resistance by increasing the processing efficiency of the substrate.

HIV Gag processing is highly regulated and results in the ordered generation of mature viral proteins [34,40–42]. Processing of the NC/p1 cleavage site is considered to be among the slowest events, which may be determined by its unusual amino acid sequence as compared to other HIV cleavage sites. Site-directed mutagenesis has demonstrated that particular changes at the P1 position of this cleavage site accelerated the rate of processing [43]. Apparently, the rate of cleavage is regulated by the use of suboptimal amino acids in the cleavage sites and can be significantly enhanced. The in vitro experiments that we have performed demonstrated the selection of NC/p1 cleavage site substitutions (A431V at the P2 position, and the K436E and I437V/T at the more distant P4′ and P5′ positions, respectively). We have shown that all these amino acid changes are directly associated with enhanced polyprotein processing. Western blot analysis demonstrated not only increased processing of the intermediate NC/p6 product but also enhanced overall polyprotein processing. Apparently, changing the kinetics of one of the rate-limiting steps (NC/p1) impacts cleavage at the other sites. Preliminary data indicate that the replicative capacity of the 436E+437T cleavage site variant is severely reduced as compared to wild-type HIV-1, indicating that enhanced Gag cleavage may come at a price. Further research is warranted to investigate the particular role of the individual cleavages and their interaction.

Little is known regarding the exact role of the distant P4′ and P5′ positions in the actual cleavage process. They are not positioned in the substrate-binding pocket of the enzyme, and it is most likely that the increased processing we observed is caused by structural changes in the Gag polyprotein, altering the conformation of the substrate region. The A431V substitution observed in our in vitro selection experiments has been described before, both in vitro and in vivo [14–16,18,36,38,44]. This mutation affects the P2 position of the cleavage site and thus is in direct contact with the enzyme's substrate-binding pocket. We and others demonstrated that changing the alanine to a valine, which is more common at this P2 position in other cleavage sites, results in enhanced cleavage at NC/p1 by wild-type protease (this study; [45]). This was structurally confirmed by the demonstration that wild-type protease can bind the wild-type NC/p1 cleavage site less well than the A431V-mutated cleavage site [46]. Moreover, increased processing was also demonstrated in the background of primary drug-resistance mutations in the protease sequence at codons 46, 82, 84, and 90 [45].

A clinical trial using a boosted PI (lopinavir with ritonavir) has demonstrated virological failure in 20% of antiretroviral therapy–naive patients [23]. This failure could not be attributed to the development of traditional phenotypic or genotypic PI resistance. Although therapy compliance may be partly responsible, further studies are warranted to investigate if increased cleavage could explain this observation. In addition, there are a number of studies indicating that in the case of boosted lopinavir there are unexplained failures in a setting where noncompliance is practically ruled out as the cause of failure. This has become most obvious in the kaletra monotherapy studies presented at the XVI International AIDS Conference in Toronto, Canada (August 2006) [47–49] . In all of these studies a proportion of patients fail virologically. Again, the viruses of most of these patients lack mutations in the viral protease, suggesting that alternative mutations may play a role.

The current introduction into clinical practice of other boosted PI regimens (such as darunavir) may further increase the selection of resistant HIV variants employing an alternative resistance mechanism. This is supported by the observation that in vitro selection experiments using this inhibitor demonstrated phenotypic resistance that could not be explained by resistance mutations in the viral protease sequence [21]. Moreover, recent data demonstrate that part of the in vitro observed resistance could be explained by *gag* mutations, including a cleavage site mutation at codon 437 [50].

Our findings may have direct implications for drug development and the monitoring of patients on a PI-failing regimen. When novel drugs are being developed, we suggest that one should evaluate whether this alternative resistance mechanism provides an alternative escape route for the virus. When evaluating a patient failing a PI-containing regimen using either a phenotypic or a genotypic approach, it is important to include the region containing the changes we observed, not only to identify resistant viruses with a wild-type protease sequence, but also because such changes may enhance resistance in the presence of well-known protease mutations.

It remains to be determined in future clinical studies whether and to what extent cleavage site mutations contribute to virological failure. We speculate that the frequency will depend on the classical variables determining treatment response, such as compliance, drug interaction, protease levels, and virological factors such as genetic barrier and the level of resistance associated with the various protease resistance mutations.

In conclusion, we have identified a novel mechanism of PI resistance involving increased polyprotein processing due to primary mutations in the natural substrate; these mutations were observed both in vitro and in vivo. Further studies are required to determine to what extent cleavage site mutations may explain virological failure during PI therapy.

## Supporting Information

Figure S1 Analysis of Gag Polyprotein Processing Western blot analysis of virus particles obtained by ultracentrifugation of cell culture supernatants from 293T cells transfected with the respective recombinant virus plasmids (wild-type or 436E+437T) in the absence of inhibitor or in the presence of 50, 100, 200, or 500 nM of RO033-4649. Protein detection was performed following incubation with a specific antibody against CA.
(37 KB JPG)

Table S1 Nucleotide Sequence of Primers Used for Amplification and Sequencing of HIV-1 (28 KB XLS)

## Abbreviations

CA: capsid
NC: nucleocapsid
PI: protease inhibitor
